# Genetic predispositions moderate the effectiveness of tobacco excise taxes

**DOI:** 10.1371/journal.pone.0259210

**Published:** 2021-11-05

**Authors:** Eric A. W. Slob, Cornelius A. Rietveld

**Affiliations:** 1 MRC Biostatistics Unit, School of Clinical Medicine, University of Cambridge, Cambridge, United Kingdom; 2 Department of Applied Economics, Erasmus School of Economics, Erasmus University Rotterdam, Rotterdam, The Netherlands; 3 Erasmus University Rotterdam Institute for Behavior and Biology, Erasmus University Rotterdam, Rotterdam, The Netherlands; University of South Florida, UNITED STATES

## Abstract

**Background:**

Tobacco consumption is one of the leading causes of preventable death. In this study, we analyze whether someone’s genetic predisposition to smoking moderates the response to tobacco excise taxes.

**Methods:**

We interact polygenic scores for smoking behavior with state-level tobacco excise taxes in longitudinal data (1992-2016) from the US Health and Retirement Study (*N* = 12,058).

**Results:**

Someone’s genetic propensity to smoking moderates the effect of tobacco excise taxes on smoking behavior along the extensive margin (smoking vs. not smoking) and the intensive margin (the amount of tobacco consumed). In our analysis sample, we do not find a significant gene-environment interaction effect on smoking cessation.

**Conclusions:**

When tobacco excise taxes are relatively high, those with a high genetic predisposition to smoking are less likely (i) to smoke, and (ii) to smoke heavily. While tobacco excise taxes have been effective in reducing smoking, the gene-environment interaction effects we observe in our sample suggest that policy makers could benefit from taking into account the moderating role of genes in the design of future tobacco control policies.

## Introduction

Tobacco use is the leading preventable cause of death in the world, causing over 7 million deaths per year [1]. In the United States, over 480,000 deaths per year are attributable to smoking [2]. Tobacco use has been shown to be quite addictive and hence, quitting is often a tough battle characterized by heavy withdrawal symptoms [3]. As a prime instrument to influence smoking behavior, the US government imposes excise taxes on tobacco. Over the past 50 years, the median price of cigarettes has increased from 0.30$ per pack up to 5.70$ [2]. In the same period, consumption per capita decreased from 4, 000 to about 1, 000 cigarettes per year. Although this decrease cannot entirely be explained by the increase in tobacco excise taxes in this period, because for example public awareness about the detrimental effects of smoking also increased in this period, there is convincing evidence about the effectiveness of raising tobacco excise taxes for reducing smoking in the population [4–6]. However, the decrease in smoking consumption has stalled in the past 20 years [7].

A possible explanation for the stabilizing smoking prevalence in the US may be that individuals differ in their responsiveness to tobacco excise taxes. For example, studies have shown that demand elasticities for tobacco differ between males and females [8] and across ethnicities [9]. Moreover, behavioral preferences such as risk aversion [10, 11] and someone’s health status influence smoking behavior [12–14]. There is also clear evidence that heavy smokers react differently to tobacco excise taxes than less heavy smokers [15], although the precise mechanisms explaining these elasticity differences are not known. In the present study, we analyze whether someone’s genetic predisposition to smoking moderates the response to tobacco excise taxes.

Several studies have shown that the heritability of smoking behavior ranges between 31–60% [16], indicating that genes explain a considerable proportion of the variation in smoking in a population. Recent large-scale genetic association studies have found more than 500 genetic variants underlying the heritable variation in smoking behavior [17, 18]. These genetic variants are primarily expressed in biological systems that affect reward processing and addiction [18]. These genetic variants thus underlie biological systems (e.g., stress system responsivity) that make a person more or less reactive to environmental conditions [19]. Earlier research has already shown that the heritability of smoking is lower in states with relatively high excise taxes on tobacco and in states with greater controls on cigarette advertising and vending machines [20], and Fletcher [21] shows that individuals carrying a particular genetic variant respond differently to excise tobacco taxes than those not carrying this genetic variant. Such a gene-environment (G×E) interaction may explain why certain individuals smoke while others do not when tobacco excise taxes are high.

However, a follow-up study in a different sample by Fontana [22] analysing the same genetic variant could not replicate the G×E interaction identified by Fletcher [21]. Despite the different sample profiles, Fontana suggests that the results of Fletcher might be due to population stratification. Population stratification entails an association between the prevalence of certain genes and environmental conditions, such as cultural and social norms, in subpopulations in the analysis sample [23]. Besides, recent studies have shown that the predictive power of individual genetic variants is limited, often below 0.02% for behavioral outcomes including smoking [24]. Hence, low statistical power may be another reason for why Fontana [22] could not replicate the results of Fletcher [21].

To deal with the limited predictive power of genetic variants, methods have been developed to combine multiple genetic variants into a composite genetic measure. The most commonly adopted approach is the construction of so-called polygenic scores (PGSs) [25]. To construct a PGS, all genetic variants in a sample are summed up in a weighted fashion in which each weight is proportional to the strength of the association between the genetic variant and an outcome variable as estimated in a genome-wide association study (GWAS) [26]. For example, a recent study shows that PGSs currently explain about 4% of the variance in smoking behavior [18]. A PGS not only makes one well-powered for out of sample prediction, but also enables more powerful G×E interaction analysis. However, by using PGSs, Fontana [22] shows that the interaction between someone’s genetic predisposition (as captured by the PGSs for educational attainment and smoking intensity) and tobacco excise taxes is insignificant in a model explaining the intensity of tobacco consumption.

The present study adopts the same approach to study G×E interactions in smoking behavior as Fontana [22] takes, but we are able to extend this earlier study in four ways. First, whereas Fontana [22] employs PGSs for educational attainment and smoking intensity, we use a set of PGSs more directly related to smoking behavior. That is, we use PGSs specifically constructed for smoking initiation, smoking intensity and smoking cessation. Second, we use PGSs that are more predictive for smoking behavior. The predictive power of PGSs is positively depending on the sample size of the GWAS which results are used to construct the PGS [25]. We use PGSs based on the recent results of the “GWAS & Sequencing Consortium of Alcohol and Nicotine use” (GSCAN) [18] which were obtained in a sample of *N* > 1, 1 million individuals (∼15× more individuals than in the GWAS on smoking behavior that Fontana [22] used to construct PGSs). Third, through the inclusion of additionally genotyped individuals (∼12,000 vs. ∼8,500) as well as non-genetic data from the three most recent waves of data collection from the US Health and Retirement Study, our analyses are better powered. As such, we have higher chances of detecting significant G×E interaction effects. Fourth, whereas Fontana [22] focusses on the intensity of smoking only, we analyze the extensive margin (smoking vs. not smoking), the intensive margin (number of cigarettes smoked per day), and smoking cessation (smoking continuation vs. smoking cessation). As such, we provide a more complete analysis of smoking behavior.

Establishing a G×E interaction is often complicated by the fact that individuals with a certain genetic predisposition may self-select into certain environments [27]. Possible bias from such a G×E correlation is often a concern when interpreting the results of earlier G×E interaction studies on smoking [28]. In this study, we deal with gene-environment correlation by exploiting exogenous variation in the level of tobacco excise rates across states and years. While those with a strong genetic predisposition to smoking may be expected to be less responsive to changes in tobacco excise taxes, Boardman [20] shows that genetic effects on smoking are less pronounced in restrictive environments (i.e., where tobacco excise taxes are relatively high). A restrictive environment may thus cushion genetic susceptibility to smoking [19], perhaps through effects on the processing of rewards: the monetary rewards of not smoking increase when tobacco prices increase. In line with this mechanism, our empirical results suggest that individuals with a high genetic propensity for smoking respond relatively strongly to changes in tobacco excise taxes: those with a high genetic predisposition to smoking are less likely to smoke when tobacco excise taxes are relatively high. If smoking, those with a high genetic predisposition to smoking consume lower amounts of tobacco in states with higher tobacco excise taxes. In our sample, we do not find a significant G×E interaction effect on smoking cessation.

Our study mainly contributes to two streams of literature. First, we enrich the literature analyzing smoking behavior and responses to tobacco excise taxes [8–15, 20] by overcoming limitations of earlier studies analysing G×E interactions on smoking [21, 22]. Second, we contribute to an emerging literature on G×E interactions exploiting *exogenous* variation in environments that addresses how the environment moderates the effect of genetic variants, and vice versa [28–33]. These studies stress that the analysis of exogenous variation in environments is key to overcome bias from gene-environment correlation when estimating G×E interactions.

## Data

The data used in this study are derived from the US Health and Retirement Study (HRS) [34]. The HRS is a longitudinal survey consisting of approximately 20,000 individuals who were surveyed biennially since 1992. The respondents in the survey are a representative sample of Americans over age 50 and their spouses. The HRS aims to analyze the health and behavior of individuals approaching or just after retirement. Therefore, the dataset includes information about for example work status, pension plans, income, health insurance, physical health and functioning, cognitive functioning, and health behaviors including drinking and smoking [35]. From 2006 onwards, the HRS started to collect genetic data from their respondents. In the present study, we exploit data collected in the waves from 1992 up to 2016 (13 waves in total) which have been harmonized by the RAND Corporation (RAND HRS Longitudinal File 2016 V2). In addition to these publicly available data, we use restricted HRS data on state of residence of the respondents. Use of this combination of HRS data for this study has been approved by our local institutional review board (ERIM:NE2019–15).

### Smoking behavior

The main outcomes in the present study are three measures of smoking behavior. The first (binary) variable is based on the question ‘Do you smoke cigarettes now?’, and equals 1 if an individual is currently smoking and 0 otherwise. The second (continuous) variable is the response to the question ‘About how many cigarettes or packs do you usually smoke in a day now?’. This question is only asked to individuals who are currently smoking, and it is set to 0 in case an individual does not smoke. Responses are converted to the average number of cigarettes smoked per day. The third (binary) variable measures smoking cessation, and is constructed using our first smoking variable. It equals 1 if an individual smokes at time (interview wave) *t* − 1 but not at time *t*, and 0 if an individual smokes at time *t* − 1 and *t*. This approach allows for the inclusion of observations from individuals who quit smoking more than once during the period of observation.

### Tobacco excise taxes

The Tax Burden on Tobacco dataset [7] provides us information about the tax levied by the state on each purchased pack of cigarettes (based on the state and federal tax in each year). We converted these nominal prices to real prices using the Consumer Price Index from the US Bureau of Labor Statistics [36], using 1991 as the base year. These data were merged with the HRS data using the restricted data on the state the HRS respondent currently lives in. As the HRS contains biennial survey data, we use the tax levied in the year prior to each survey. The reason for this is that the Tax Burden on Tobacco dataset contains the tax applied in a year based on fiscal years ending June 30. As a result, it can be that an individual is interviewed by the HRS before changes in the excise tax are effective in a state. For consistency with prior studies and to facilitate the interpretation of effects as proportional changes in consumption, the tax levels are logarithmically transformed [21, 37].

### Polygenic scores

Polygenic scores are used to analyze whether the response to tobacco excise taxes is moderated by someone’s genetic predisposition to smoking. Most genetic differences across individuals in a population can be attributed to single-nucleotide polymorphisms (SNPs). A SNP is a location in the DNA strand at which two different nucleotides can be present in the population. For each SNP, an individual’s genotype is coded as a 0, 1 or 2, depending on the number of reference nucleotides present. Individuals who inherited the same nucleotide from each parent are called homozygous for that SNP (and have genotype 0 or 2), while individuals who inherited different nucleotides are called heterozygous (and have genotype 1). PGSs reflect the combined additive influence of SNPs on a particular outcome.

To construct a PGS, SNPs are summed up in a weighted fashion. The weights reflect the strength of the relationship between a SNP and the outcome of interest, as estimated in a GWAS. In a GWAS, for each SNP the following model is estimated:

$$\begin{array}{c} y_{i}=\mu +\gamma_{m}g_{im}+\delta z_{i}+\nu_{i}, \end{array}$$ (1)

where *y*_*i*_ is the outcome of interest for individual *i*, *μ* is an intercept, *γ*_*m*_ is the additive effect of SNP *g*_*im*_, *z*_*i*_ is a vector of control variables (e.g., sex and age), and *ν*_*i*_ is the residual term. Using the effect size estimates *γ*_*m*_ from Eq 1, the PGS is constructed as:

$$\begin{array}{c} G_{i}=\sum_{m=1}^{M}\gamma_{m}g_{im}, \end{array}$$ (2)

where *G*_*i*_ represents the value of the PGS for individual *i*, *M* is the total number of SNPs included in the construction of the PGS, *γ*_*m*_ is the additive effect size of SNP *m* taken as estimated in the GWAS and *g*_*im*_ is the genotype of individual *i* at locus *m* (measured as 0, 1 or 2).

The HRS provides PGSs for public distribution based on the results of several recently conducted large-scale GWASs [38]. In this study, we use three PGSs to measure someone’s genetic predisposition to smoking behavior. The first PGS is based on the results of a GWAS on *smoking initiation (ever smoked vs. never smoked)*, and measures someone’s genetic predisposition to start smoking. The second PGS is based on the results of a GWAS with the *number of cigarettes smoked per day* as dependent variable. As such, the second PGS reflects someone’s genetic predisposition to heavy smoking. Lastly, we have a PGS that is based on the results of a GWAS on *smoking cessation (currently smoking vs. smoking formerly)*. This PGS captures someone’s genetic predisposition to being able to stop smoking after having started smoking. Hence, the first PGS reflects the genetic predisposition to smoking on the extensive margin, the second one reflects the genetic predisposition to smoking on the intensive margin, and the third one for smoking cessation.

The weights used to construct the PGSs were obtained from the GSCAN consortium [18]. In total, approximately 1.4 million SNPs were used to construct the PGSs [38]. We use the PGSs constructed for individuals of European ancestry in the sample, because the GSCAN analyses were also restricted to individuals of this ancestry. To facilitate an easy interpretation of the results, the PGSs are standardized such that they have mean 0 and a standard deviation of 1 in the analysis sample. Higher values reflect a higher genetic predisposition to smoking behavior. For smoking cessation, a higher score reflects a higher chance to stop smoking.

### Covariates

For comparability purposes, the choice of individual level control variables is based on the studies by Fletcher [21] and Fontana [22]. We include an individual’s sex as a covariate, to control for differences between males and females. Furthermore, we include an individual’s birth year to account for possible age specific differences in smoking behavior and we add birth year squared to account for possible non-linearities in the age effects. We account for the socio-economic status of the respondent by including individual income (as imputed by the RAND Corporation [39], in real terms using 1991 as base year) and years of education (self-reported by participants) in the model.

Although Fontana [22] controls in his models for the change in health status, we abstain from it because of possible endogeneity issues [14]. Compared to Fletcher’s model [21], we do not control for race/ethnicity because we restrict our sample to individuals of recent European ancestry. This is a commonly used restriction in genetic studies and also recommended by the genotyping center, because this restriction pre-empts possible bias from population stratification [40]. That is, correlations between allele frequencies and environmental factors across subpopulations in the analysis sample. To deal with more subtle forms of population stratification in the analysis sample, we include the first 10 genetic principal components of the genetic relationship matrix as control variables [38]. The genetic relationship matrix includes pairwise genetic relationships between individuals in the sample as estimated using SNPs. It has been shown that the inclusion of principal components solves the problem of subtle population stratification adequately in the HRS [23].

Finally, we include state dummies and wave dummies to account for heterogeneity across states and time.

## Methods

Use of the HRS data for this study has been approved by the local institutional review board (Internal Review Board for Non-Experimental Research of the Erasmus Research Institute of Management, Erasmus University Rotterdam, reference number IRB NE 2019–15 Eric Slob).

To test for the presence of an effect of the interaction between someone’s genetic predisposition to smoking behavior and tobacco excise taxes on smoking outcomes, we use a moderation framework for each of our three outcome variables. The baseline regression for explaining whether an individual is currently smoking is given by:

$$\begin{array}{c} S_{ist}=\alpha_{0}+\alpha_{1}{\text{Tax}}_{st}+\alpha_{2}G_{i}+\alpha_{3}G_{i}{\text{Tax}}_{st}+\alpha_{4}X_{ist}+S_{s}+D_{t}+\varepsilon_{ist}, \end{array}$$ (3)

where *S*_*ist*_ is a binary variable indicating whether individual *i* residing in state *s* in year *t* is currently smoking or not, Tax_*st*_ represents the tobacco excise taxes in state *s* in year *t*, and *G*_*i*_ is the value of the PGS for individual *i*. *X*_*ist*_ represents the vector of individual-level control variables. The *α*’s represent the corresponding effect size estimates for these variables. The vectors *S*_*s*_ and *D*_*t*_ contain state and year fixed effects, respectively. Lastly, *ε*_*ist*_ denotes the error term. Despite the binary nature of *S*_*ist*_, we estimate the model using linear regression to make the interpretation of the coefficient more straightforward and to avoid the difficulties surrounding the estimation of interaction effects in non-linear models. However, we note that we obtain qualitatively similar results when using a logit specification.

The response to tobacco excise taxes in terms of tobacco consumption is estimated by:

$$\begin{array}{c} C_{ist}=\beta_{0}+\beta_{1}{\text{Tax}}_{st}+\beta_{2}G_{i}+\beta_{3}G_{i}{\text{Tax}}_{st}+\beta_{4}X_{ist}+S_{s}+D_{t}+\tau_{ist}, \end{array}$$ (4)

where *C*_*ist*_ denotes the number of cigarettes smoked per day by individual *i* at time *t* in state *s*. The other variables are the same as in Eq 3. In this equation, the *β*’s are the effect size estimates and *τ*_*ist*_ is the residual term. We estimate this model both in the full sample and in the subsample of smokers, because non-smokers are not likely to start smoking when tobacco excise taxes increase.

Finally, we analyze smoking cessation using discrete-time survival models. Allison [41] shows that such survival models can be operationalized by using regression models for binary dependent variables. Therefore, we perform a binary logistic regression to explain the binary variable for smoking cessation. Importantly, this model can deal with right-censored observations, such as individuals who are still smoking in our final year of observation (2016). This model can be written as:

$$\begin{array}{c} logit(E_{ist})=\zeta_{0}+\zeta_{1}{\text{Tax}}_{st}+\zeta_{2}G_{i}+\zeta_{3}G_{i}{\text{Tax}}_{st}+\zeta_{4}X_{ist}+S_{s}+D_{t}+\phi_{ist}, \end{array}$$ (5)

where *E*_*ist*_ denotes whether individual *i* in state *s* stopped smoking between time *t* − 1 and time *t*. In this regression, the *ζ*’s are the effect size estimates and *ϕ*_*ist*_ is the residual term. In our models, we do not use clustered standard errors as recommended by Allison [41]. As a result of the inclusion of wave dummies in the models, the hazard rate (the probability that an individual stops smoking between time *t* − 1 and time *t* given that (s)he has not yet done so at *t* − 1) is assumed to be different in each of the 13 waves in the sample.

## Results

**Fig 1:**
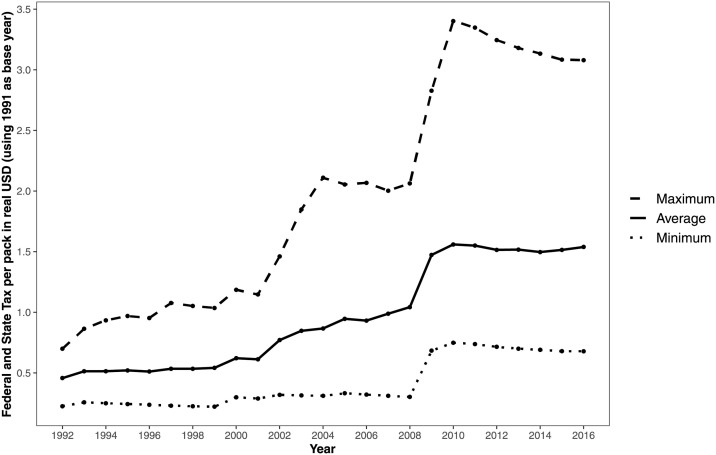
Real tobacco excise taxes (1991 $) levied per pack of 20 cigarettes in US states (1992–2016).

For our analyses, we merged the genotyped individuals of European ancestry (*N* = 12, 090) with the geographic information from the restricted HRS data on state of residence. From these individuals, 32 cannot be included in the analyses because of missing data on smoking behavior. Table 1 contains the descriptive statistics of the analysis sample, both for the full sample and the subsample of current smokers. In total, our analysis sample comprises 105,959 observations from 12,058 distinct individuals. Time-invariant variables are constant over the waves of data collection, but time-variant variables can take different values over time. In the full sample, there are more females than males and the mean birth year is 1941. There are small differences between the full sample and the subsample of smokers with respect to birth year, years of education, income, and marital status. Not surprisingly, the means of the PGSs for smoking behavior as well as the smoking prevalence and the average number of cigarettes smoked per day are relatively high in the subsample of current smokers. The smoking cessation variable is only constructed for those who smoked in at least one of the interview waves, and is 0 by definition for all current smokers. Fig 1 shows that tobacco excise taxes gradually increase over time (in real terms) and that they differ considerably across states.

**Table 1:** Descriptive statistics analysis sample.

	Full sample		Subsample of current smokers	
**Time-invariant variables**	Mean	Std. Dev.	Mean	Std. Dev.
Female	0.58	0.49	0.59	0.49
Birth year	1938	10.33	1943	9.57
Years of education	13.20	2.52	12.43	2.34
*PGS*_Smoking initiation_	0.000	1.000	0.28	1.02
*PGS*_Smoking intensity_	0.000	1.000	0.14	0.99
*PGS*_Smoking cessation_	0.000	1.000	0.12	0.96
**Time-variant variables**	Mean	Std. Dev.	Mean	Std. Dev.
Currently smoking	0.14	0.34	1.00	0.00
Cigarettes smoked per day	2.32	7.26	17.10	11.66
Smoking cessation	0.15	0.36	0.00	0.00
Income (×$1,000)	11.66	29.93	11.84	22.43
Married	0.69	0.46	0.61	0.49
Individuals	12,058		2,642	
Observations	105,959		14,385	

*Notes*: Std. Dev. = Standard deviation. The analysis sample comprises 12,058 individuals from which 2,642 are currently smoking in at least one interview wave.

Table 2 present the results of the model explaining whether an individual is currently smoking (the extensive margin). Column 1 shows that higher state-level tobacco excise taxes are negatively associated with the dependent variable, and that the PGS for smoking initiation is positively associated with an individual’s current smoking status. Both these results are in line with expectations. In terms of effect sizes, an increase of excise taxes by 1% reduces the likelihood of smoking by about 0.07 percentage points, and an increase of one standard deviation in the PGS increases the likelihood of smoking by about 4 percentage points.

**Table 2:** Results of the regressions explaining an individual’s current smoking status.

	(1)	(2)	(3)
Log(Tax)	-0.066***	-0.066***	-0.000
	(0.005)	(0.005)	(0.005)
*PGS*_Smoking initiation_	0.038***	0.036***	0.036***
	(0.003)	(0.004)	(0.004)
Log(Tax) × *PGS*_Smoking initiation_		-0.012***	-0.012***
		(0.003)	(0.003)
Female		-0.012***	-0.012***
		(0.003)	(0.003)
Birth year	0.024	0.022	-0.101
	(0.077)	(0.077)	(0.074)
Birth year^2^	-0.000	-0.000	0.000
	(0.000)	(0.000)	(0.000)
Income (×$1,000)	-0.000***	-0.000***	-0.000***
	(0.000)	(0.000)	(0.000)
Years of education	-0.016***	-0.016***	-0.016***
	(0.001)	(0.001)	(0.001)
Married	-0.080***	-0.080***	-0.088***
	(0.006)	(0.006)	(0.006)
State & Wave dummies	No	No	Yes
Observations	105,959	105,959	105,959
Individuals	12,058	12,058	12,058
*R* ^2^	0.0780	0.0783	0.0938

*Notes*: Standard errors in parentheses (clustered by state and individual); Coefficients for the constant term and the principal components are not reported, but available upon request from the authors;

* *p* < 0.05,

** *p* < 0.01,

*** *p* < 0.001.

In Column 2, the interaction term between the state-level tobacco excise taxes and the PGS for smoking initiation has been added to the model. This interaction term is significantly negative, indicating that those with a high genetic predisposition to smoking respond relatively strongly to tobacco excise taxes. Column 3 shows that upon inclusion of state and wave fixed effects, the coefficient for tobacco excise taxes becomes insignificant. This change can be explained by the fact that tobacco taxes within a state increase in a monotonic fashion over time. These dynamics are absorbed by the state and wave dummies in this model (such absorption is commonly observed in the literature, see, e.g., [6, 21, 22]). However, the interaction term between the PGS and tobacco excise taxes remains statistically significant in Column 3.

**Fig 2:**
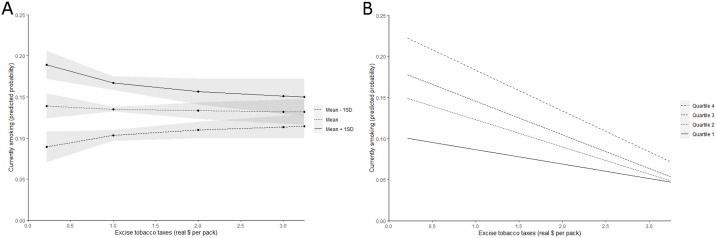
The relationship between excise tobacco taxes and the likelihood of smoking. Panel A: The relationship between tobacco excise taxes and the predicted probability of smoking, as evaluated at the mean (+/- 1 Standard Deviation (SD)) of the polygenic score for smoking initiation. Gray areas represent 95% confidence intervals. Panel B: The relationship between tobacco excise taxes and the predicted probability of smoking in each quartile of the distribution of the polygenic score for smoking initiation.

Panel A of Fig 2 visualizes the gene-environment interaction effect of Column 3 in Table 2). It can be seen that those with a higher genetic predisposition to smoking tend to respond stronger to changes in tobacco excise taxes than those with a lower PGS value. We also note that smoking remains prevalent at any point in the distribution of the genetic predisposition to smoking. Panel B of Fig 2 depicts a graphical representation of the G×E interaction effect based on regression analyses within each quartile of the PGS distribution (the corresponding regression results for each quartile are available in S1 File). In each quartile, there is a negative relationship between the level of excise tobacco taxes and the predicted probability of currently smoking. The slopes of the relationships again suggest that individuals with the highest genetic predisposition to smoking (Quartile 4) respond stronger to tobacco excise taxes than those with a lower genetic predisposition to smoking.

Table 3 presents the results of the regressions explaining someone’s smoking intensity (the intensive margin, in terms of cigarettes per day). In Column 1 (Full sample) and Column 4 (Subsample of current smokers), tobacco excise taxes are significantly negatively associated with the number of cigarettes smoked per day. In terms of effect sizes, an increase of excise taxes by 1% reduces cigarette consumption by 0.016 cigarettes per day in the full sample, and 0.035 cigarettes in the sample of current smokers. The PGS is again predictive of smoking behavior (one standard deviation increase in the PGS leads to an increase in consumption of 0.36 cigarettes per day in the full sample and an increase of 1.07 cigarettes per day in the subsample of current smokers). Column 2 shows that the G×E interaction effect is significantly negative in the full sample. This suggests that within the full sample, individuals with a high value for the PGS tend to consume lower amounts of tobacco when tobacco excise tax are high.

**Table 3:** Results of the regressions explaining an individual’s smoking intensity.

	Full sample			Subsample of current smokers		
	(1)	(2)	(3)	(4)	(5)	(6)
Log(Tax)	-1.585***	-1.587***	-0.024	-3.490***	-3.437***	-0.158
	(0.115)	(0.115)	(0.151)	(0.396)	(0.388)	(0.448)
*PGS*_Smoking intensity_	0.358***	0.318***	0.319***	1.072***	0.961***	0.927***
	(0.053)	(0.048)	(0.048)	(0.161)	(0.156)	(0.154)
Log(Tax) × *PGS*_Smoking intensity_		-0.204**	-0.205**		-0.376	-0.377*
		(0.059)	(0.060)		(0.191)	(0.181)
Female	-0.901***	-0.902***	-0.980***	-3.316***	-3.314***	-3.718***
	(0.093)	(0.093)	(0.096)	(0.358)	(0.357)	(0.364)
Birth year	1.937	1.916	-0.958	13.93*	13.62*	11.28
	(1.561)	(1.569)	(1.503)	(6.665)	(6.659)	(6.499)
Birth year^2^	-0.000	-0.000	0.000	-0.004*	-0.003*	-0.003
	(0.000)	(0.000)	(0.000)	(0.002)	(0.002)	(0.002)
Income (×$1,000)	-0.005**	-0.005**	-0.009**	0.001	0.001	-0.013
	(0.002)	(0.002)	(0.003)	(0.006)	(0.006)	(0.008)
Years of education	-0.337***	-0.337***	-0.321***	-0.320***	-0.319***	-0.289**
	(0.023)	(0.023)	(0.021)	(0.089)	(0.089)	(0.085)
Married	-1.429***	-1.430***	-1.623***	-0.363	-0.358	-0.938**
	(0.121)	(0.121)	(0.121)	(0.296)	(0.296)	(0.297)
State & Wave dummies	No	No	Yes	No	No	Yes
Observations	105,930	105,930	105,930	14,356	14,356	14,356
Individuals	12,058	12,058	12,058	2,634	2,634	2,634
*R* ^2^	0.058	0.058	0.075	0.066	0.066	0.105

*Notes*: Standard errors in parentheses (clustered by state and individual); Coefficients for the constant term and the principal components are not reported, but available upon request from the authors;

* *p* < 0.05,

** *p* < 0.01,

*** *p* < 0.001.

**Fig 3:**
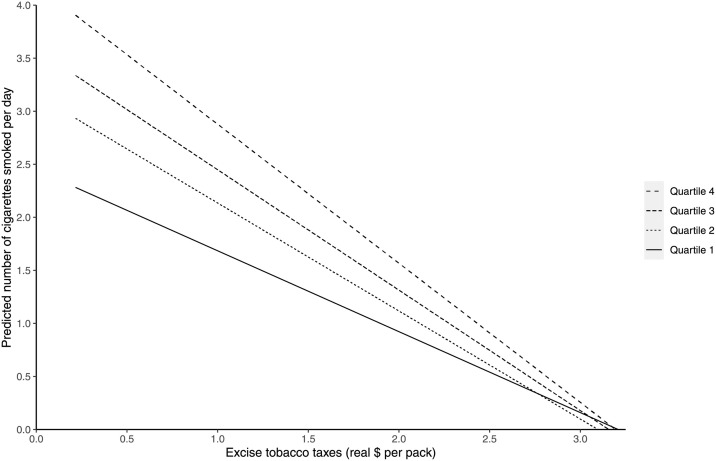
The relationship between excise tobacco taxes and the amount of tobacco consumption in each quartile of the distribution of the polygenic score for smoking intensity.

When comparing the results in the full sample with those in the subsample of current smokers, we observe that the effect sizes are relatively large in the latter subsample. The estimates suggest that current smokers are reacting more strongly to changes in tobacco excise taxes. This can be explained by the fact that smokers are able to reduce their smoking intensity, whereas in the full sample the non-smokers are not likely to change their smoking behavior in response to increases in tobacco excise taxes (i.e., to start smoking). We observe that the interaction term in this subsample is not significant at the 5% level (Column 5). When adding state and wave dummies (Column 6), the interaction effect becomes significant. This change suggests that the (borderline) insignificant finding in Column 5 is due to the relatively small sample size. Fig 3 illustrates these findings based on regressions analyses within quartiles of the PGS distribution (the corresponding regression results for each quartile are available in S2 Table in S1 File). In the full sample, the relationship between excise tobacco taxes and tobacco consumption is most negative in the fourth quartile (individuals with the highest genetic predisposition to heavy smoking). When we perform the same analysis in the subsample of current smokers (see S1 File), we observe a similar pattern.

In Table 4, the results of the model explaining smoking cessation are shown. Column 1 shows that tobacco excise taxes are significantly associated with smoking cessation. In terms of effect size, doubling excise taxes changes the odds of smoking cessation by 8.7%. We observe that the PGS is also predictive for smoking cessation (a one standard deviation increase in the PGS changes the odds of smoking cessation by 7.3%). The G×E interaction term is insignificant, both in the model without state and wave dummies (Column 2) and in the model with these control variables (Column 3). Like in Tables 2 and 3, the effect of tobacco excise taxes becomes insignificant upon inclusion of the state and wave dummies (Column 3).

**Table 4:** Results of the (logit) regressions explaining an individual’s decision to cessate smoking.

	(1)	(2)	(3)
Log(Tax)	0.120*	0.126*	-0.128
	(0.049)	(0.049)	(0.125)
*PGS*_Smoking cessation_	0.076*	0.090**	0.102**
	(0.034)	(0.033)	(0.034)
Log(Tax) × *PGS*_Smoking cessation_		-0.059	-0.060
		(0.041)	(0.040)
Female	-0.106	-0.106	-0.083
	(0.058)	(0.058)	(0.058)
Birth year	-0.230	-0.256	-0.184
	(0.784)	(0.791)	(0.818)
Birth year^2^	0.000	0.000	0.000
	(0.000)	(0.000)	(0.000)
Income (×$1,000)	-0.002	-0.002	-0.000
	(0.002)	(0.002)	(0.001)
Years of education	0.054***	0.054***	0.047**
	(0.015)	(0.015)	(0.015)
Married	0.116	0.118	0.160*
	(0.068)	(0.068)	(0.071)
State & Wave dummies	No	No	Yes
Observations	12,842	12,842	12,832
Individuals	2,599	2,599	2,595
Pseudo-*R*^2^	0.0072	0.0074	0.0187

*Notes*: Standard errors in parentheses (clustered by state); Coefficients for the constant term and the principal components are not reported, but available upon request from the authors;

* *p* < 0.05,

** *p* < 0.01,

*** *p* < 0.001.

Due to perfect prediction, 10 observations (4 individuals) were dropped in Column 3.

In sum, our empirical results show that the interaction between an individual’s genetic predisposition to smoking and state-level tobacco excise taxes significantly impacts whether someone smokes or not (the extensive margin) and the amount of tobacco consumption (the intensive margin), but not smoking cessation. In the next subsection, we present robustness checks scrutinizing the validity of these inferences.

### Robustness checks

Our first robustness checks concerns the use of a one year lag for the tobacco excise taxes in our models. Since the HRS contains biennial survey data, we use the tax levied in the year prior to each survey in the main models. The prime reason is that the Tax Burden on Tobacco dataset contains the tax applied in a year based on fiscal years ending June 30. Nevertheless, when using current taxes (like, e.g., [6]), we obtain similar results. That is, the G×E interaction term in Table 2 (Model 3) remains exactly −0.012 (SE = 0.003) with *p* < 0.001. The same term in Table 3 (Model 3) changes from −0.205 (SE = 0.059) with *p* < 0.01 to −0.208 (SE = 0.060) with *p* < 0.01. Finally, the interaction term in Table 3 (Model 6) changes from −0.377 (SE = 0.181) with *p* < 0.05 to −0.372 (SE = 0.185) with *p* = 0.053. Thus, our results are not driven by using one year lags for tobacco excise taxes in the main models.

As a second robustness check, we investigate to what extent our main results are driven by not accounting for relevant factors such as anti-smoking sentiment. Changes in tobacco excise taxes may covary with anti-smoking sentiment, and therefore we need to verify that the G×E effects we estimate can indeed be attributed to changes in tobacco excise taxes. Using data from from the Tobacco Use Supplement to the Current Population Survey (TUS-CPS) [42], we find that controlling for the presence of clean indoor air laws (CIALs) and anti-smoking sentiment barely changes the estimated G×E interactions. Additionally controlling for interaction effects between the CIAL and anti-smoking sentiment measures and the PGSs for smoking behavior introduces severe multicollinearity in the models, but does not change our main inferences. Finally, we amended our models with state-specific time trends to control for all sorts of time-varying effects that are difficult to capture otherwise. Again, we generally observe that the G×E interaction effects remain similar in size and significance. Taken all these results into account, we conclude that controlling for relevant anti-smoking policies or sentiments does not meaningfully change our main inferences. More information about these robustness checks can be found in S1 File.

Our third robustness check follows the proposal of Keller [43] to include interaction terms between the PGSs and the control variables to overcome residual confounding in G×E models. This proposal is particularly relevant for gene-environment studies exploiting *endogenous* environments, while our study exploits an *exogenous* environment. Nevertheless, we find that the estimated G×E interactions remain similar in sign and significance when amending our models with these additional control variables. If anything, the magnitude of the G×E interaction terms in our main models somewhat increase. That is, the interaction term in Table 2 (Model 3) changes from −0.012 (SE = 0.003) with *p* < 0.001 to −0.017 (SE = 0.003) with *p* < 0.001. The same term in Table 3 (Model 3) changes from −0.205 (SE = 0.059) with *p* < 0.01 to −0.293 (SE = 0.061) with *p* < 0.001. Finally, the interaction term in Table 3 (Model 6) changes from −0.377 (SE = 0.181) with *p* < 0.05 to −0.641 (SE = 0.199) with *p* < 0.01. Therefore, we conclude that our results are not driven by residual confounding related to interactions between the PGSs and the control variables.

We check for selection bias in our fourth robustness check. Due to the adverse health effects of tobacco consumption, we expect (heavy) smokers to pass away on a relatively young age. This could lead to bias in our estimates due to selection in the analysis sample, because HRS respondent may not have survived until genetic data collection. Domingue and colleagues [44] find that adverse risks associated with smoking behavior can bias the findings in G×E studies. The magnitude of the bias varies by trait, but the direction of the bias is towards the null because the genotypes that are linked to increased mortality are less likely to be observed. This suggests that, if anything, our G×E estimates are conservative. When excluding individuals born before 1930 from the analysis sample, the G×E interaction term in Table 2 (Model 3) changes from −0.012 (SE = 0.003) with *p* < 0.001 to −0.013 (SE = 0.003) with *p* < 0.001. The same term in Table 3 (Model 3) changes from −0.205 (SE = 0.059) with *p* < 0.01 to −0.211 (SE = 0.076) with *p* < 0.01. Finally, this term in Table 3 (Model 6) changes from −0.377 (SE = 0.181) with *p* < 0.05 to −0.355 (SE = 0.179) with *p* = 0.053. Based on the similarity of the estimates, we conclude that mortality selection is not driving our main results.

Finally, we reran our models using household income instead of individual income as control variable. While we followed Fontana [22] for our model setup, household income may better capture the available socio-economic resources than individual income. We find, however, that our results remain qualitatively unchanged in this robustness check. That is, the G×E interaction term in Table 2 (Model 3) changes from −0.012 (SE = 0.003) with *p* < 0.001 to −0.012 (SE = 0.004) with *p* < 0.001. The same term in Table 3 (Model 3) remains exactly −0.205 (SE = 0.059) with *p* < 0.01. Finally, this term in Table 3 (Model 6) changes from −0.377 (SE = 0.181) with *p* < 0.05 to −0.379 (SE = 0.192) with *p* = 0.055.

## Discussion and conclusion

The present study shows that someone’s genetic predisposition and tobacco excise taxes not only impact smoking behavior additively, but also that someone’s genetic predisposition to smoking behavior moderates the impact of tobacco excise taxes on tobacco consumption (both along the extensive and intensive margin). However, this G×E interaction does not have a meaningful impact on smoking cessation. Our robustness checks provide evidence supporting these results.

Although earlier studies suggest that older individuals react less strongly to changes in excise tobacco taxes than younger individuals [45, 46], our findings suggest that even in a relatively old sample of individuals tobacco excise taxes are an effective policy instrument that can be used to reduce tobacco consumption. Studies drawing on representative samples of the adult population find price elasticities for the amount of tobacco consumption of around −0.20 [37, 47] in models with state and wave fixed effects, but Maclean and colleagues [6] estimate this elasticity to be −0.03 in the HRS (data until 2008). We find an even smaller elasticity of −0.01 in a model without the PGS for smoking intensity but with state and wave dummies. This difference is partially driven by the inclusion of data from after 2008 in our models. By including these additional waves of data collection, we also cover for instance the relatively large increases in tobacco excise taxes due to the Children’s Health Insurance Program Reauthorization Act of 2009 (see also Fig 1). We find an elasticity of −0.02 when using only HRS data until 2008. The remaining difference might be due to the use of different subsamples. Our analysis sample is restricted to individuals of recent European ancestry, but smoking behavior is known to differ across ethnicities [9]. To deal with population stratification bias, the restriction to one ancestry group is necessary but it also leads to missing out on an important source of heterogeneity in our models.

Our findings not only suggest that tobacco excise taxes are an effective method to reduce tobacco consumption in the population, but also that this reduction in particularly large amongst individuals with a high genetic predisposition to smoking. Although Fletcher [21] was the first one to show that individuals carrying a certain genetic variant respond differently to increases in tobacco excise taxes, Fontana [22] suggested that this finding could have been driven by bias from population stratification. Based on a weighted combination of multiple (approximately 1.4 million SNPs) genetic variants, i.e., a PGS, in the present study we do find again a significant interaction effect along the extensive and intensive margin for smoking. The sample restriction to individuals of European ancestry and the inclusion of principal components makes that the present findings are not likely to be driven by (subtle forms of) population stratification. However, in contrast with the findings of Fontana [22], we do find a significant impact of the interaction between the genetic predisposition to heavy smoking and excise taxes on someone’s smoking behavior. We believe this is the result of us using PGSs with higher predictive power which are also more closely related to the smoking outcomes analyzed. Moreover, our analyses were also more powerful because of our larger analysis sample both in terms of individuals and observations per individual. As such, the present findings contribute to the literature analysing heterogeneity in smoking behavior [15] by highlighting genes as an important factor moderating the response to tobacco excise taxes.

The PGSs for smoking primarily relate to biological systems that affect addiction and to systems that affect reward processing [18]. If the PGSs for smoking would capture the “addiction” mechanism only, we may have expected those with a strong genetic predisposition to be least responsive to changes in tobacco excise taxes. Boardman [20] already shows that genetic effects on smoking are less pronounced in restrictive environments (e.g., where tobacco excise taxes are relatively high). A restrictive environment may thus cushion genetic susceptibility to smoking [19], perhaps through effects on rewards processing because the monetary rewards of not smoking increase when tobacco prices increase. Our individual-level results also show that those with a high genetic propensity for smoking respond relatively strongly to changes in tobacco excise taxes, but even in the environments (i.e., US states) with the highest tobacco excise taxes the smoking prevalence is highest amongst those with the highest genetic predisposition to smoking (see, e.g., Fig 2). If smoking, those with a high genetic predisposition to smoking consume lower amounts of tobacco in states with high tobacco excise taxes. Therefore, it is likely that the G×E interactions we estimated primarily reflect the “rewards” mechanism.

Nevertheless, the interaction between the genetic predisposition to smoking and tobacco excise taxes does not significantly impact smoking cessation in our sample. The insignificance of this interaction could be driven by the relatively small sample size in these analyses. However, it may also somewhat reflect the age composition of the sample. The HRS samples individuals aged over 50 years and their spouses, and several smokers in the sample may already have been smoking for the largest part of their life and the effect of further increases in tobacco excise taxes on smoking behavior may no longer be dependent on their genetic predisposition. Still, we provide evidence that tobacco excise taxes and the genetic predisposition to smoking cessation do additively impact the decision to remain smoking. Thus, overall, our findings are largely in line with Boardman [20] showing that environmental circumstances such as state policies (including taxation policies) moderate the effect of genes on smoking. Moreover, they provide an explanation for why Nesson [15] finds that heavy smokers react differently to tobacco excise taxes than less heavy smokers.

Our *gene-environment* interaction study goes beyond Boardman’s earlier *heritability-environment* interaction study [20] by using individual-level molecular genetic information to analyze smoking behavior. As such, it allows for analyses evaluating the effectiveness of policies on the individual-level [48, 49]. Although this does not imply that it is possible to accurately predict individual-level behavioral outcomes [50], from a policy perspective our findings clearly suggest that there is genetic heterogeneity in response to tobacco excise taxes. Individuals with a high genetic predisposition towards smoking respond stronger to tobacco excise taxes compared to individuals with a lower genetic predisposition. Thus, large increases in tobacco excise taxes lower smoking behavior more among those with a high genetic predisposition to smoking than could be expected based on the effect of excise taxes alone. This is relevant to consider for policy makers, because while they cannot change the genetic make-up of individuals they can influence the environments individuals are exposed to.

More generally, G×E studies allow for the analysis of treatment effect heterogeneity and may help to identify environments (which can be shaped by policy) that cushion genetic disadvantage [21, 29] or that make individuals thrive [51]. PGSs can therefore be used to improve our understanding of the distributional consequences of policy interventions [52]. For example, tobacco taxation is a policy tool credited for a significant reduction in the rate of smoking [4–6]. Yet, many smokers seem resistant to this policy. If G×E studies would reveal that those who keep smoking even if tobacco excise taxes are high are solely individuals with a genetic predisposition to smoking, one might be limited to pharmacotherapy or pharmacogenetics to further reduce smoking in the population. In our study, we find that tobacco excise taxes are an effective method to reduce smoking in the population but also that smoking remains prevalent at any point in the distribution of the genetic predisposition to smoking. If follow-up studies would show that those who keep smoking have for example experienced adverse (childhood) environments, further prevention efforts could be targeted at modifiable characteristics in these environments. Importantly, policy makers can incorporate results from such G×E analyses without knowing the actual genotypes of specific individuals.

## Supporting information

S1 File Supplementary analyses. (PDF)
