# Supplementary material for: Genetic predispositions moderate the effectiveness of tobacco excise taxes
Source: PLoS One. 2021 Nov 5;16(11):e0259210. doi: 10.1371/journal.pone.0259210 (PMC8570524; doi:10.1371/journal.pone.0259210)
Supplement: S1 File — (PDF) [file pone.0259210.s001.pdf]

S1 File. Supplementary analyses

Regressions in subsamples based on quartiles of the polygenic score distribution

In our main analyses presented in the main text, we focus on the multiplicative linear interaction between the polygenic scores (PGSs) for smoking behavior and excise tobacco taxes. We find significant gene-environment (G×E) interactions in the models explaining an individual's current smoking status and in the models explaining the intensity of smoking. In order to investigate possible non-linear interactions, we here provide regressions results obtained in subsamples based on quartiles of the distribution of the PGSs.

Table S1 presents the results of the regressions explaining an individual's current smoking status, and it shows that the effect of tobacco excise taxes gradually increases over the quartiles. These results suggest that the linear interaction term fits the data adequately. Table S2 presents the results of the regressions explaining the amount of tobacco consumption. Again, we find that the effect of tobacco excise taxes increases over the four quartiles, but the increase in effect size flattens somewhat off in the fourth quartile. Fig S1 visualizes the results obtained in the subsample of current smokers.

Fig. S1. The relationship between excise tobacco taxes and the amount of tobacco consumption in each quartile of the distribution of the polygenic score for smoking intensity (subsample of current smokers).

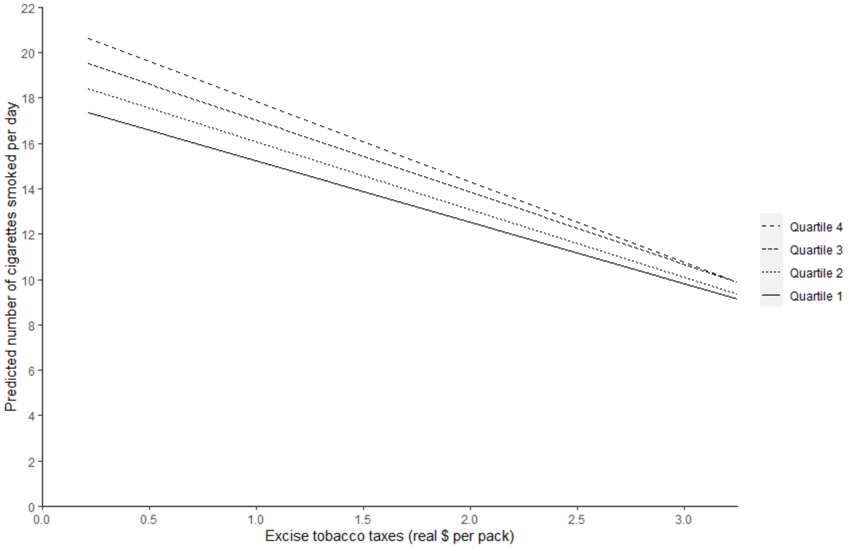

Regressions controlling for anti-smoking policies and sentiment

As a robustness check, we discuss in the main text to what extent our main results are driven by not accounting for relevant factors such as anti-smoking sentiment. Changes in tobacco excise taxes may covary with anti-smoking sentiment, and therefore we need to verify that the G×E effects we estimate can indeed be attributed to changes in tobacco excise taxes. We conclude that controlling for relevant anti-smoking policies or

Table S1. Results of the regressions explaining an individual's current smoking status in subsamples based on quartiles of distribution of the polygenic score for smoking initiation.

	(1)	(2)	(3)	(4)
Log(Tax)	-0.041***	-0.064***	-0.076***	-0.083***
	(0.007)	(0.008)	(0.007)	(0.007)
Female	-0.002	-0.022*	-0.022	-0.059***
	(0.007)	(0.009)	(0.012)	(0.014)
Birth year	0.212*	-0.039	0.046	-0.059
	(0.102)	(0.149)	(0.124)	(0.138)
Birth year^2	-0.000*	0.000	-0.000	0.000
	(0.000)	(0.000)	(0.000)	(0.000)
Income (×\$1,000)	-0.000**	-0.000***	-0.000*	-0.000
	(0.000)	(0.000)	(0.000)	(0.000)
Years of education	-0.010***	-0.017***	-0.015***	-0.023***
	(0.002)	(0.003)	(0.002)	(0.002)
Married	-0.062***	-0.073***	-0.082***	-0.097***
	(0.012)	(0.012)	(0.013)	(0.010)
Observations	26,491	26,493	26,497	26,478
Individuals	3,012	3,000	3,020	3,026
R^2	0.0462	0.0638	0.0664	0.0946

Notes: Standard errors in parentheses (clustered by state and individual); Coefficients for the constant term and the principal components are not reported, but available upon request from the authors; * $p < 0.05$, ** $p < 0.01$, *** $p < 0.001$.

sentiments does not meaningfully change our main inferences. Here, we provide the empirical results supporting this conclusion.

For this robustness checks, we employ data from the Tobacco Use Supplement to the Current Population Survey (TUS-CPS). These data are available at `https://cancercontrol.cancer.gov/brp/tcrb/tus-cps/questionnaires-data`. Whereas other datasets containing clean indoor air laws (CIALs) and anti-smoking sentiment measures do not fully cover the time period of our analysis sample, the advantage of the TUS-CPS dataset is that it covers the period 1992-2019. As such, it covers the same time period as the HRS. Using the TUS-CPS data, we constructed three state-level measures:

- CIAL measure 1 (CIAL 1): The per-state percentage of work places with an official policy that restricts smoking (based on the question whether the place of work of the respondent has an official policy that restricts smoking in any way);
- CIAL measure 2 (CIAL 2): The per-state percentage of work places with a complete or a partial smoking ban at work (two variables, based on the question whether smoking at the place of work of the respondent is not allowed in any indoor public or work areas, or not allowed in some/all indoor public or work areas);
- Anti-smoking sentiment (AS): The per-state percentage of individuals thinking that smoking in indoor work areas should not be allowed at all (based on the question whether smoking in indoor work areas should not be allowed at all).

Since the TUS-CPS questionnaires were not sent out every year, we imputed some per-state averages using linear interpolation. Still, the sample sizes in the robustness

Table S2. Results of the regressions explaining an individual's smoking intensity in subsamples based on quartiles of the distribution of the polygenic score for smoking intensity.

	Full sample				Subsample of current smokers			
	(1)	(2)	(3)	(4)	(5)	(6)	(7)	(8)
Log(Tax)	-1.221***	-1.424***	-1.817***	-1.892***	-2.426***	-3.600***	-3.544***	-4.084***
	(0.111)	(0.145)	(0.185)	(0.199)	(0.576)	(0.549)	(0.494)	(0.642)
Female	-0.560***	-0.508**	-1.155***	-1.386***	-2.703**	-2.827***	-3.454***	-3.937***
	(0.152)	(0.170)	(0.207)	(0.300)	(0.896)	(0.646)	(0.661)	(0.627)
Birth year	2.975	-0.438	4.903	0.682	-1.513	16.43	12.50	18.98
	(2.422)	(2.243)	(2.817)	(3.417)	(12.12)	(10.13)	(11.22)	(9.415)
Birth year^2	-0.001	0.000	-0.001	-0.000	0.000	-0.004	-0.003	-0.005
	(0.000)	(0.001)	(0.001)	(0.001)	(0.003)	(0.003)	(0.003)	(0.002)
Income (×$1,000)	-0.004**	-0.005	-0.008**	-0.004	0.013	0.001	-0.002	0.002
	(0.001)	(0.002)	(0.003)	(0.003)	(0.016)	(0.011)	(0.011)	(0.007)
Years of education	-0.244***	-0.361***	-0.366***	-0.371***	-0.366	-0.322*	-0.311	-0.336**
	(0.030)	(0.041)	(0.039)	(0.047)	(0.186)	(0.133)	(0.194)	(0.107)
Married	-1.096***	-1.064***	-2.022***	-1.575***	0.610	-0.0621	-1.508*	-0.217
	(0.226)	(0.213)	(0.283)	(0.222)	(0.642)	(0.468)	(0.685)	(0.647)
Observations	26,489	26,478	26,493	26,470	2,806	3,520	3,898	4,132
Individuals	3,018	2,995	3,036	3,009	535	635	696	768
R^2	0.0402	0.0568	0.0703	0.0560	0.0452	0.0615	0.0861	0.0733

Notes: Standard errors in parentheses (clustered by state and individual); Coefficients for the constant term and the principal components are not reported, but available upon request from the authors; * $p < 0.05$, ** $p < 0.01$, *** $p < 0.001$.

analyses based on the TUS-CPS data are slightly smaller because we lack information about these additional variables in 1991. In our analyses we use the one year lag of tobacco excise taxes, and by doing the same for these additional variables we effectively lose the 1992 data in our analysis sample. However, we note that we obtain qualitatively similar results when using current values rather than one year lags of the TUS-CPS variables.

As a first robustness check, we reran our main models using these measures (lagged by one year) as additional control variables. The results for the different analyses are shown in Table S3. For the model explaining someone's current smoking status, the coefficient of the G×E interaction term barely changes upon the inclusion of the control variables for CIALs and anti-smoking sentiment. The same holds for the models explaining an individual's smoking intensity.

As a second robustness check, following the first one, we also added interaction effects between the CIAL and anti-smoking sentiment measures (lagged by one year) and the PGSs for smoking behavior to the models. The results of these analyses are also shown in Table S3. Compared to the results of the first robustness check, we observe that the signs of the interaction terms remain the same. However, their magnitudes generally decrease while their standard errors increase. As a result, most interaction terms are no longer statistically significant at $p < 0.05$. Besides the slight drop in sample size in these analyses (as compared to the original models), these changes seem due to severe multicollinearity that is introduced in these models with the additional interaction terms. That, the correlation between the tobacco excise taxes and anti-smoking sentiment is as high as 0.69 with CIAL 1, respectively 0.68 and −0.62 with the two variables of CIAL 2, and 0.68 with AS. Therefore, we focus in these models on the direction and magnitude of the coefficients.

Third, we amend our models with state-specific time trends. These trends capture

Table S3. The estimated G×E interaction effects between tobacco excise taxes and polygenic scores (PGSs) for smoking behavior in the models without and with control variables for the presence of clean indoor air laws (CIALs) in the state and anti-smoking sentiment (AS) in the state.

	Original model	Original model + CIAL 1	Original model + CIAL 2	Original model + AS
	Models not including additional interactions with the PGSs			
Current smoking status	-0.012***	-0.011***	-0.011**	-0.011**
(full sample)	(0.003)	(0.003)	(0.003)	(0.003)
Smoking intensity	-0.205**	-0.188**	-0.188**	-0.195**
(full sample)	(0.060)	(0.061)	(0.061)	(0.061)
Smoking intensity	-0.377*	-0.388*	-0.397*	-0.420*
(current smokers only)	(0.181)	(0.188)	(0.186)	(0.185)
	Models including additional interactions with the PGSs			
Current smoking status	-0.012***	-0.008	-0.007	-0.006
(full sample)	(0.003)	(0.005)	(0.005)	(0.005)
Smoking intensity	-0.205**	-0.100	-0.055	-0.106
(full sample)	(0.060)	(0.078)	(0.086)	(0.078)
Smoking intensity	-0.377*	-0.414	-0.508*	-0.520*
(current smokers only)	(0.181)	(0.219)	(0.235)	(0.234)

Notes: Standard errors in parentheses (clustered by state and individual); Coefficients for the other variables in the models are not reported, but available upon request from the authors; * $p < 0.05$, ** $p < 0.01$, *** $p < 0.001$.

all sorts of time-varying effects that are difficult to capture otherwise. In Table S4 we present the estimated effect of the G×E interaction in the models where we allow for state specific time trends. We observe that the G×E interaction effects remain similar in size and significance, except in case of the smoking intensity analysis in the subsample of current smokers. Here, the p-value changes to 0.055.

Table S4. The estimated G×E interaction effect between tobacco excise taxes and polygenic scores (PGSs) for smoking behavior in the models without and with state-specific time trends.

	Original model	Original model + state-specific time trends
Current smoking status	-0.012***	-0.012***
(full sample)	(0.003)	(0.003)
Smoking intensity	-0.205**	-0.201*
(full sample)	(0.060)	(0.078)
Smoking intensity	-0.377*	-0.392
(current smokers only)	(0.181)	(0.198)

Notes: Standard errors in parentheses (clustered by state and individual); Coefficients for the other variables in the model are not reported, but available upon request from the authors; * $p < 0.05$, ** $p < 0.01$, *** $p < 0.001$.

Taking into account the results of these three robustness checks, we conclude that controlling for relevant anti-smoking policies or sentiments does not meaningfully change our main inferences.
